# Evaluation of anion exchange resin for sorption of selenium (IV) from aqueous solutions

**DOI:** 10.1186/s13065-024-01356-3

**Published:** 2025-01-09

**Authors:** A. El-Tantawy, E. M. Abu Elgoud, S. E. A. Sharaf El-Deen

**Affiliations:** 1 Nuclear Fuel Technology Department, Hot Laboratories Center, Egyptian Atomic Energy Authority, P.O. 13759, Cairo, Egypt; 2 Nuclear Fuel Chemistry Department, Hot Laboratories Center, Egyptian Atomic Energy Authority, P.O. 13759, Cairo, Egypt; 3 Nuclear Chemistry Department, Hot Laboratories Center, Egyptian Atomic Energy Authority, P.O. 13759, Cairo, Egypt

**Keywords:** Amberlite IRA-400 Cl^−^, Se(IV), Sorption, Isotherm and kinetics, Desorption and regeneration

## Abstract

In this work, selenium (IV) ions were adsorbed from aqueous solutions by the strongly basic anion exchange resin Amberlite IRA-400. The morphology of the resin before and after Se(IV) sorption was investigated using different techniques such as energy dispersive X-ray spectroscopy (EDX), Fourier transform infrared spectroscopy (FTIR), and scanning electron microscopy (SEM). To determine the ideal sorption conditions, a batch approach was used to examine the variables affecting Se(IV) sorption performance, including pH, shaking time, adsorbent dosage, initial metal ion concentration, and temperature. The results showed the optimal parameters for the highest percentage of selenium (80.25%) at an initial concentration of 100.0 mg L^−1^, pH 3.0, the adsorbent dosage of 10.0 mg, and the shaking time of 60.0 min. According to the experimental findings, the sorption process was satisfactorily explained by the pseudo-second-order kinetic model. The maximum adsorption capacity at pH 3.0 was 18.52 mg g^−1^, and the adsorption rather well followed the Langmuir adsorption isotherm. Moreover, exothermic and spontaneous sorption reaction was the result of thermodynamic properties (negativity of both ΔG° and ΔH°). The adsorption phase's random distribution of the resin-solution interface is indicated by the positive value of ΔS^o^. Finally, the desorption study was performed using different concentrations of desorbing agents; HNO_3_, HCl, and sodium acetate. The results illustrated that the effective desorbing agent was 1.0 mol L^-1^ HNO_3_, with desorption efficiency reaching about 96.4%. Finally, the Amberlite IRA-400 demonstrated excellent adsorption–desorption behavior over five times, suggesting that the Amberlite IRA-400 could be an effective candidate for the sorption of Se(IV) from several metal ions that occur in fission products.

## Introduction

The management of radioactive waste is a significant concern for nuclear energy and different industries that employ radioactive materials. The removal of long-lived radionuclides is a major challenge to the safe and efficient disposal of radioactive waste [1–5]. One such radionuclide is selenium, which is found in fission products and radioactive waste due to its presence in nuclear fuel cycle processes and the decay of other radioactive isotopes. Selenium is a naturally occurring element with several isotopes, but the most common radioactive isotope is selenium-79. This isotope has a half-life of approximately 3.27 × 105 years, making it a persistent and potentially hazardous component of radioactive waste [6, 7]. If not properly managed, selenium-79 can pose long-term risks to human health and the environment. Selenium is significantly applied in numerous products, including semiconductors, pesticides, and photosensitive materials, and thus can be emitted into the environment through sources such as agricultural runoff and industrial effluent [8, 9]. Additionally, it is also utilized in the production of ceramics, catalysts, and the medical field due to it being an important element for living organisms [10]. Selenium (IV) has been removed from aqueous solutions using a variety of techniques, such as solvent extraction [11], co-precipitation [12], membrane separation [13], adsorption [14, 15], and multiple evaporation processes [16]. One of the most promising approaches for removing selenium is the adsorption process, which is considered to be inexpensive, easy to operate, and efficient for the removal of Se(IV) from aqueous solutions. Numerous types of adsorbents, such as hematite-modified magnetic nanoparticles [17], phlogopite and calcite surfaces [18], zirconium and iron oxides [19], aluminum oxide-coated sand [20], chitosan-clay composite [21], iron-coated GAC [22], nanocrystalline hydroxyapatite [23], iron oxide impregnated CNTs [24], Mg-FeCO_3_ loaded cellulose fiber [25], Al/Si and Fe/Si Coprecipitates [26], poly(1,8-diaminonaphthalene) [27], and iron oxide/hydroxide-based nanoparticles sol (NanoFe) [28] have been applied for the removal of Se(IV) from aquatic solutions. Amberlite IRA-400 is known for its strong ion exchange capacity, making it ideal for many applications such as the removal of selenium and other metal ions. The Amberlite IRA-400 resin already contains highly effective functional groups, like quaternary ammonium groups, which provide excellent ion exchange capacity for removing elements like selenium from aqueous solutions. These functional groups allow the resin to perform efficiently without the need for any modifications. Therefore, there is no necessity to modify the resin, as it is already designed to effectively achieve the treatment process. The objective of this research was to assess the efficiency of an anion exchange resin (Amberlite IRA-400) in the sorption of Se(IV) from aqueous solutions. Amberlite IRA-400 resin was analyzed using FT-IR, SEM, and EDX. Through the study of the isothermal, kinetic, thermodynamic, and regeneration characteristics of sorption, the mechanism of sorption was further clarified.

## Materials and methods

### Materials

Analytical-grade chemicals and double-distilled water were utilized to prepare all chemical solutions. BDH Chemical Company supplied Amberlite IRA-400 anion exchange resin. The key features of Amberlite IRA-400 are displayed in Table 1. Sodium hydroxide, hydrochloric acid, and selenium dioxide (SeO_2_) were purchased from Sigma-Aldrich.

**Table 1:** Specifications of the ion exchange resin Amberlite IRA-400

Parameters	Specification
Polymer matrix	Polystyrene DVB
Functional group	–N^+^ R3
*Ionic* form	Cl^−^
*Exchange* capacity	2.6–3 eq kg^−1^ (dry mass)
Effective size	0.3–0.9 mm
Operating temperature	80 °C (maximum)
pH range	0–14
Structure	

### Characterization of the Amberlite IRA-400 resin

Before and after sorption, the characteristics of anion exchange resin (Amberlite IRA-400) were examined using FT-IR, SEM, and EDX. Japan's JSM-6510 LA SEM model was used to identify the morphological properties of anion exchange resin. The resin spectra were examined with a Nicolet IS10 Fourier transformer infrared (FT-IR) spectrometer from Meslo, USA. A quantitative elemental analysis was performed on the Amberlite IRA-400 both before and after Se(IV) adsorption using an Oxford energy-dispersive X-ray (EDX) spectrometer (Oxford Link ISIS, Japan). The concentrations of selenium ions were determined using an atomic absorption spectrophotometer of the Model S4 Series manufactured by Thermo-electron Corporation.

### Batch experimental procedures

Using the batch equilibrium technique, the use of Amberlite IRA-400 for the sorption of Se(IV) from aquatic solutions was investigated. The following variables were optimized: pH, temperature, adsorbent dose, starting concentration, and shaking duration. For the adsorption evaluations, 0.10 g of resin and 5.0 ml (100 mg L^−1^) of Se(IV) were shaken in a shaker bath (G.F.L. 1083, Germany) at 25 °C, and the rpm was set to 250 for ion exchange or sorption studies in all batch experiments. Using an atomic absorption spectrophotometer, the initial selenium concentrations before (C_o_) and after (C_e_) sorption were calculated. The sorption percent (S%) of Se(IV) and the sorption capacity (q_o_) of Amberlite IRA-400 were calculated using the relations

1 $$S\% = \left( {\frac{{{\text{C}}_{{\text{o}}} - {\text{C}}_{{\text{e}}} }}{{{\text{C}}_{{\text{o}}} }}} \right) \times 100$$

2 $${\text{q}}_{{\text{o}}} = ({\text{C}}_{{\text{o}}} - {\text{C}}_{{\text{e}}} )\left( {\frac{{\text{V}}}{{\text{m}}}} \right) \left[ {{\text{mg}} {\text{g}}^{ - 1} } \right]$$

The initial and equilibrium concentrations (mg L^−1^) of Se(IV) in the solution are represented by C_o_ and C_e_, respectively. V is the solution volume (L), and m is the Amberlite IRA-400 resin weight (g).

### Mathematical models

Table 2 summarizes the kinetic and isotherm models that were applied. To investigate the sorption kinetics, both pseudo-first and pseudo-second-order equations were illustrated. To set the experimental data, Freundlich, Langmuir, Dubinin-Radushkevich, and thermodynamic models were utilized.

**Table 2:** Models and equilibrium isotherms for the sorption of selenium ions onto Amberlite IRA-400 resin [29–31]

Isotherm	Linear form	Plot	Slope and intercept
Langmuir	$\frac{{C}_{e}}{{q}_{e}}=\left(\frac{1}{{Q}_{o}b}\right)+\left(\frac{1}{{Q}_{o}}\right){C}_{e}$ R_L_ = 1/ 1 + bC_o_	C_e_/q_e_ versus C_e_	Slope = 1/ Q_o_ Intercept = 1/ Q_o_ b
Freundlich	$\mathit{log}{q}_{e}=\mathit{log}{K}_{f}+\frac{1}{n}\mathit{log}{C}_{e}$	log q_e_ versus log C_e_	Slope = 1**/**n Intercept = logK_f_
Dubinin–Radushkviech	$ln{q}_{e}=ln{q}_{m}-\beta {\varepsilon }^{2}$ $\varepsilon =RTln\left[1+\left(1/{C}_{e}\right)\right]$	lnq_e_ versus ${\varepsilon }^{2}$	Slope = −*β* Intercept = ln* q*_*m*_
Thermodynamic	*lnK*_d_ =(∆S°/R)−(∆H°/R)$(1 /T)$ ∆G° = −*RTlnK*_d_	lnK_d_ versus 1/T	Slope = −ΔH°/R Intercept = ΔS°/R
*Kinetic model*			
Pseudo-First-order	$\text{log}\left({q}_{e}- {q}_{t}\right)=log{q}_{e}-\frac{{K}_{1 }}{2.303} t$	Log(q_e_-q_t_) versus t	Slope = −k_1_**/**2.303 Intercept = log q_e_
Pseudo-Second-order	$\frac{\text{t}}{{q}_{t}}=\frac{1}{{K}_{2 }{q}_{e}^{2}}+\frac{t}{{q}_{e}}$	t/q_t_ versus t	Slope = 1**/**q_e_ Intercept = 1**/** K_2_ ${q}_{e}^{2}$

## Results and discussion

### Characterization of the resin

An energy-dispersive X-ray spectroscopy (EDX), scanning electron microscopy, and Fourier transform infrared (FTIR) spectroscopy were used to evaluate the resin chemical and physical characteristics.

#### Fourier transformed infrared (FTIR) spectroscopy

**Fig. 1:**
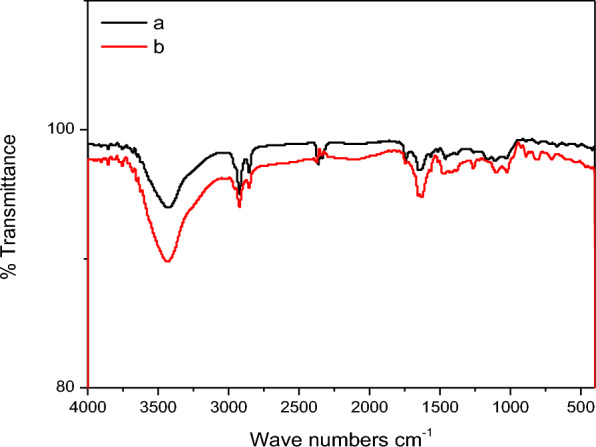
FTIR of **a** Amberlite IRA-400 resin and **b** Amberlite IRA-400 resin after sorption of Se(IV) ions

FT-IR analysis was performed to display the structure of Amberlite (IRA-400) resin both before and after the sorption of Se(IV). The resulting spectrums have been recorded and presented in Fig. 1a, b. Styrene–divinylbenzene polymeric matrixs spectra exhibit a prominent peak at 3426 cm^−1^, which may be ascribed to –OH, as illustrated in Fig. 1a. The asymmetric C−H stretching vibrations of CH_2_ and CH_3_ in CH_3_−N are responsible for the band at 2923 cm^−1^ [32]. Furthermore, many peaks are seen at 1637, 1513, 1380, 1262, 1030, and 520 cm^−1^. These could be related to the –C–C– functional groups, as well as the C=O, and C−N stretching vibrations of benzene rings [33]. The peaks at 1030 and 890 cm^−1^, respectively, are caused by the C–O and C–H bonds. Upon comparing the spectra, the FT-IR spectrum of Amberlite (IRA-400) following Se(IV) sorption is displayed in Fig. 1b, exhibiting several discernible alterations. These modifications (at 1625, 1262, and 1024 cm^−1^) could therefore be the result of Se(IV) binding to adsorbent.

#### Scanning electron microscopy (SEM)

**Fig. 2:**
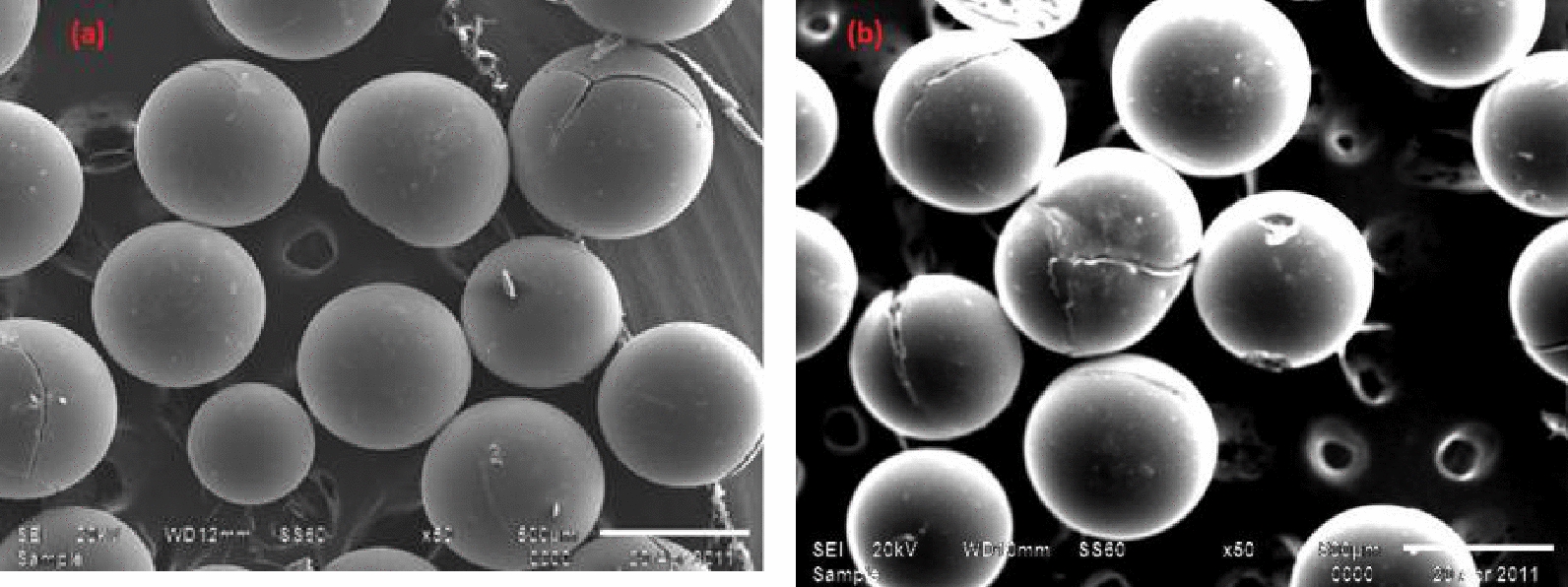
SEM images of **a** Amberlite IRA-400 resin and **b** Amberlite IRA-400 resin after sorption of Se(IV) ions

The surface morphology of Amberlite (IRA-400) before and after sorption of Se(IV) is investigated by SEM at 50X magnification, as illustrated in Fig. 2a, b. It can be seen that Amberlite (IRA-400) has smooth, spherically shaped particles. While the surface structure after the adsorption of Se(IV) showed some minor cracks in the particle surface and not changes in the shape of the particles, that illustrate approximately the stability of the resin.

#### Energy dispersive X-ray spectroscope (EDX)

**Fig. 3:**
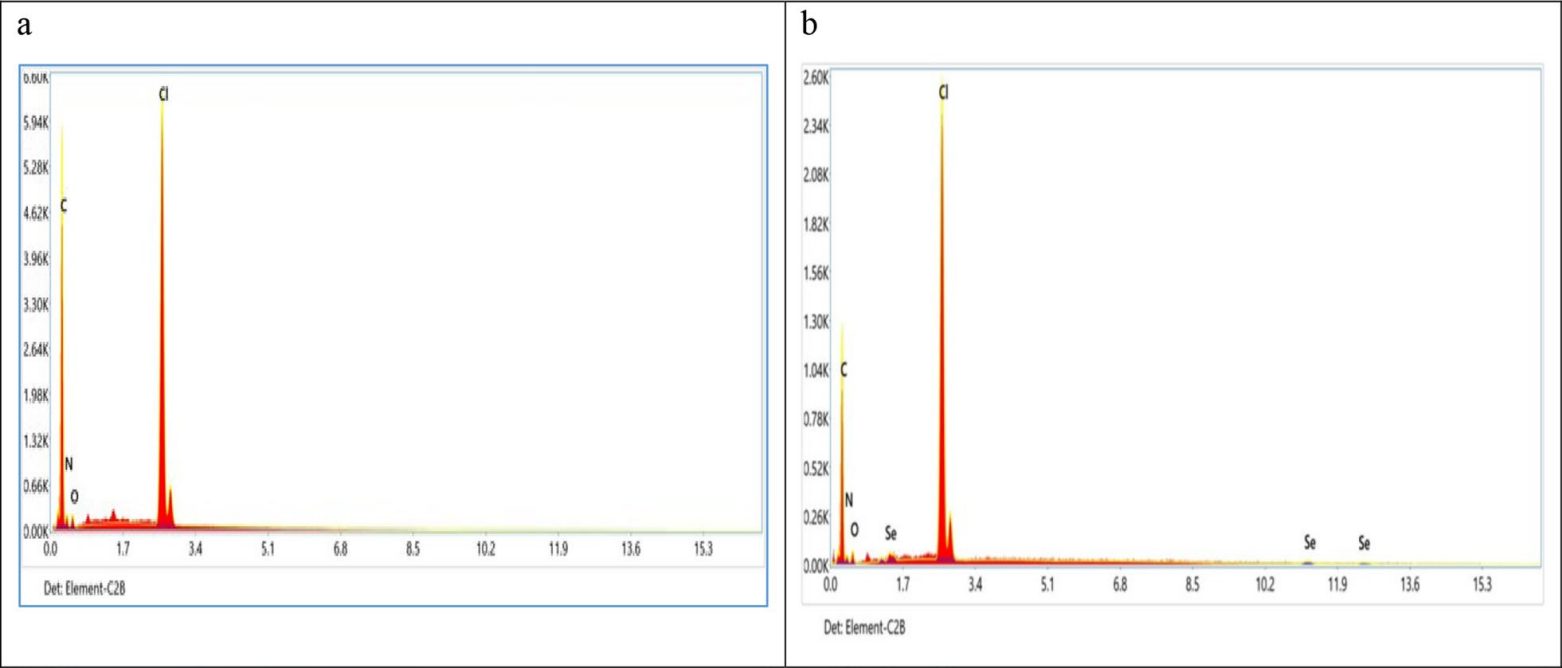
EDX of **a** Amberlite IRA-400 resin and **b** Amberlite IRA-400 resin after sorption of Se(IV) ions

Figure 3a, b displays the Amberlite (IRA-400) EDX spectrum both before and after the sorption of Se(IV). Based on the EDX examination, the main components of Amberlite (IRA-400) (Fig. 3a) include C (78.35%), N (7.39%), O (3.19%), and Cl (11.07%). Additionally, components C (74.29%), N (5.37%), O (3.79%), Cl (15.26%), and Se (1.29%) were detected in the Amberlite (IRA-400) EDX spectrum following Se(IV) adsorption (Fig. 3b).The presence of Se elements in the loaded resin was verified by the EDX characterization.

### Batch sorption studies

#### Effect of pH

**Fig. 4:**
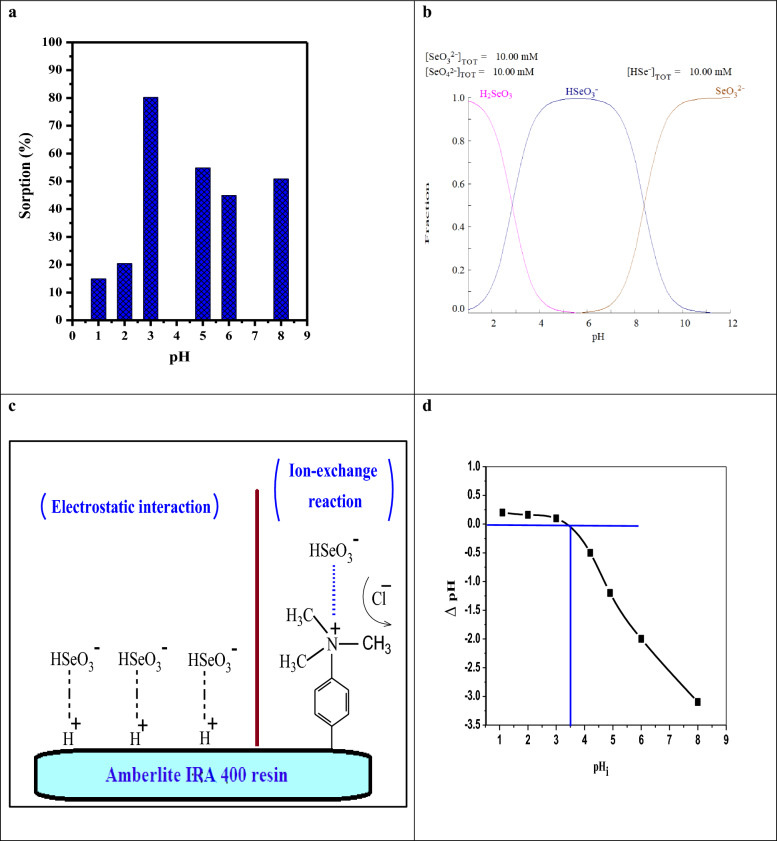
**a** pH impact on the sorption efficiency of Se(IV) from aqueous solutions, (t = 60.0 min, [Se (IV)] = 100.0 mg L^−1^, dose = 0.1 g, V = 5.0 mL, T = 25 ºC). **b** Speciation digram of Se(IV) at different pH. **c** Suggested mechanism of the electrostatic and ion-exchange reactions that occur on the surface of the Amberlite IRA-400 resin and **d** Zero point charge (pH_ZPC_) of the resin

The pH of the adsorption medium plays a significant role in affecting the ionization characteristics of the active sites on the adsorbent [31]. The effect of solution pH on the sorption percentage of Se(IV) from aqueous solution in the range of 1.0 to 8.0 was investigated, and the obtained results were presented in Fig. 4a. It was noticed that the sorption effectiveness increased from 14.92 to 80.25% as the pH increased from 1.0 to 3.0. After that, as the pH increased, the sorption percentage decreased. Using the Hydra/Medusa chemical equilibrium software, the speciation diagrams for Se(IV) are shown in Fig. 4b. From this figure, it is clear that at low pH, the predominate species of Se(IV) is selenious acid (H_2_SeO_3_). This explained the low values for the sorption percent at pH ≤ 2. Moreover, as the pH increased up to 3.5, the predominant species biselenite (HSeO_3_^−^) appeared. Based on the obtained results, at low pH values, the anionic resin used in the experiment contains positive charges (a higher number of H^+^ ions), so an electrostatic reaction occurs between the HSeO3- species and the positive charge of the resin surface. Thus, pH 3.0 was chosen for the sorption process of selenium ions in the following investigations. Furthermore, the sorption is related to the chemical composition of the resin that contains amino functionalities in its—R_3_N^+^Cl^−^ structure, which leads to an increase the sorption process at pH 3.0. This process can occur via an ion exchange reaction, as indicated in Fig. 4c [34, 35]. It was confirmed by a zero-point charge. The zero-point charge (pH_zpc_) of the resin was measured at pH 3 (Fig. 4d). The sorbent surface holds positive charges at pH less than pH_zpc_, which increases the electrostatic attraction force with biselenite (HSeO_3_^−^). The proposed mechanism is clarified in Fig. 4c**.**

#### Effect of shaking time

**Fig. 5.**
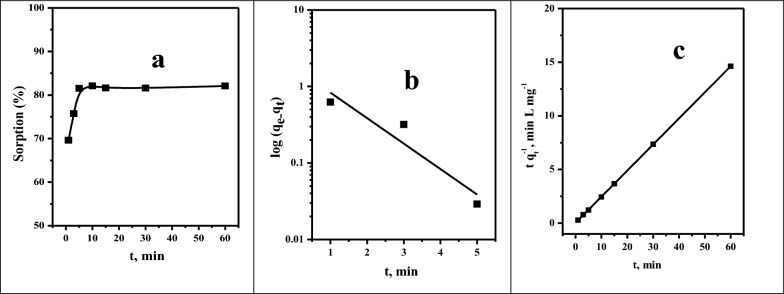
**a** shaking time impact on the sorption percentage: **b** pseudo-first-order and **c** pseudo-second-order kinetic model for Se(IV) with concentration of 100 mg L^−1^, dosage of 0.10 g, volume of 5 mL, pH 3.0, and 25 °C from aqueous solutions.

One of the most crucial factors to consider when developing a batch sorption experiment is the rate of sorption. It affects removal efficiency, contributes to contact time optimization, supports kinetics modeling, and contributes to establishing suitable experimental conditions. The impact of shaking time on selenium sorption was examined in each experiment under the following conditions: pH 3.0, initial concentration of 100.0 mg L^−1^, and adsorbent mass of 0.10 g. Figure 5a depicts the evaluation of Se(VI) sorption on the Amberlite IRA-400 resin as a shaking time in the range of 1.0–60.0 min. We observe that the sorption percentage of Se(IV) increases from 69.62 to 82.10% when the shaking time increases from 1.0 to 10.0 min. After that, the sorption percentage remained constant for 60.0 min. Two main linear kinetic models, the pseudo-first-order model and the pseudo-second-order model, have been used to explain the selenium adsorption process onto Amberlite IRA-400 resin (see Fig. 5b,c). Table 3 summarizes the correlation coefficient (R^2^) and several parameters of these kinetic models. It is noted that the pseudo-second-order is close to the unit (R^2^ = 0.999). Furthermore, the experimental result, q_e EXP_ = 4.11 mg g^−1^, and the calculated q_eCAL_ = 4.12 mg g^−1^ values match quite well. These results demonstrated that the Se(VI) adsorption on Amberlite IRA-400 is adequately explained by the pseudo-second-order model.

**Table 3:** Kinetic model parameters for the sorption of Se(IV) ([Se(IV)] = 100 mg L^−1^, pH = 3.0, V = 5.0 mL, adsorbent dosage = 0.10 g, T = 25 °C) from aqueous solutions

Kinetic	Parameters	Metal ion
		Se(IV)
Pseudo-first order	q_e_ (mg/g)	1.79
	K_1_ (g/mg.min)	0.767
	R^2^	0.809
Pseudo-second order	q_e_ (mg/g)	4.12
	K_2_ (g/mg.min)	1.76
	R^2^	0.999
q_exp_, mg/ g		4.11

#### Effect of initial selenium ion concentration

**Fig. 6:**
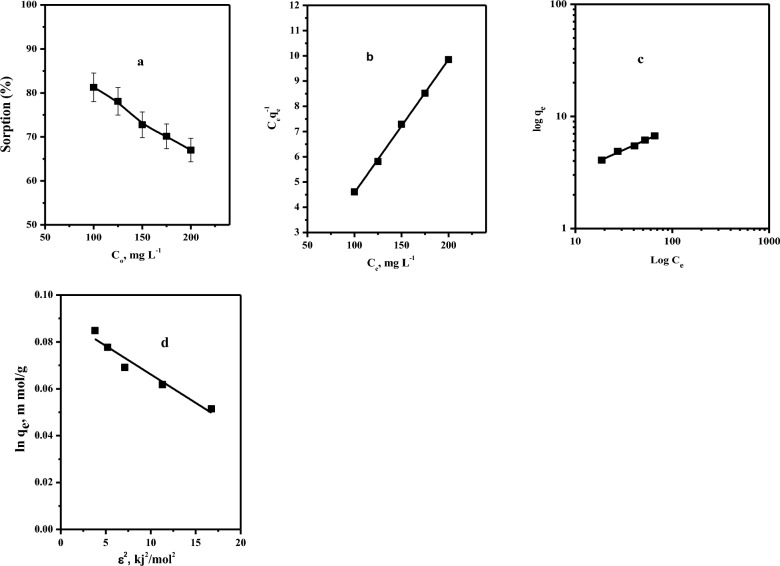
**a** Effect of initial Se(IV)ion concentration **b** Plots of linear Langmuir isotherms; **c** Plots of linear Freundlich isotherm** d** Linear Dubinin–Radushkevich isotherm on the sorption percent of Se(IV) from aqueous media. (t = 10.0 min, pH = 3.0, Dose = 0.10 g, V = 5 ml, T = 25 ºC)

Figure 6a illustrates the impact of initial metal ion concentrations on the sorption percentage of selenium. We observed that the sorption efficiency of Se(IV) decreased from 81.27 to 67.0% as the initial concentration of Se(IV) was increased from 100.0 to 200.0 mg L^-1^. These results can be attributed to the fact that, at low initial concentrations of Se(IV), the quantity of Se(IV) species in the solution is relatively small compared to the total number of available binding sites on the surface of the adsorbent. In this case, there are sufficient binding sites available to accommodate the Se(IV) species, which leads to high sorption efficiency. As more Se(IV) is added into the system at higher initial concentrations, the number of Se(IV) species in the solution increases, while the number of binding sites remains constant. As a result, the sorption efficiency will directly decrease [36, 37].

#### Sorption isotherm and modeling

To estimate the adsorption mechanism for the interaction of Se(IV) ions on the Amberlite IRA-400 surface, adsorption isotherms at equilibrium are crucial. Various linear isotherm models were utilized to evaluate the results of the experiment. Table 4 represents the values calculated from the slopes and intercepts for Se(IV) sorption obtained from Fig. 6b–d. It was shown that the Langmuir model has the highest correlation coefficient (0.999). This showed that the selenium species prefer to adsorb as monolayer adsorption onto Amberlite IRA-400 active sites.

**Table 4:** Linearized sorption isotherm parameters for Se(IV) sorption onto the Amberlite IRA-400 resin

Isotherm	Parameters	Metal ion
		Se(IV)
Langmuir	Q_o_ (mg g^−1^)	18.52
	b (ml mg^−1^)	− 0.066
	R_L_	− 0.18
	R^2^	0.99961
Freundlich	K_f_ (mg g^−1^)	1.32
	n	4.925
	R^2^	0.98745
Dubinin–Radushkevich	*q*_*m*_(mmol/g)	1.09
	*β*	0.0024
	*R*^*2*^	0.934
	E_DR_	14.34

#### Effect of adsorbent dose

**Fig. 7:**
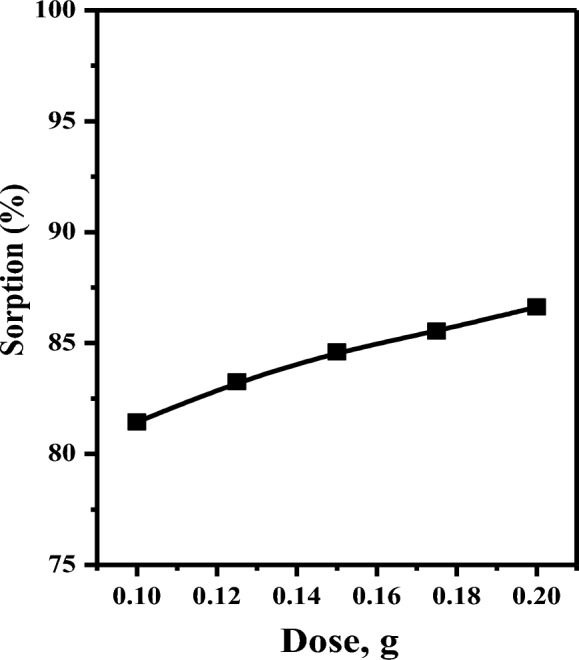
Effects of adsorbent dos on the sorption percentage of Se(IV) from aqueous solutions ([Se(IV)] = 100 mg L^−1^, V = 5 ml, pH = 3.0, t = 10.0 min, T = 25 °C)

Figure 7 illustrates the impact of Amberlite IRA-400 resin on selenium sorption efficiency in the range of 0.10–0.20 g. According to the results of the experiment, the sorption performance of Se(IV) increased from 81.44 to 86.62% when the adsorbent dosage was increased from 0.10 to 0.20 g. The observed increase in sorption can be attributed to the higher adsorbent dosage, which leads to an increased availability of active sites for the sorption of selenium species. For additional sorption investigations, 0.1 g of Amberlite IRA-400 was determined to be the optimum dosage.

#### Effect of solution temperature

**Fig. 8:**
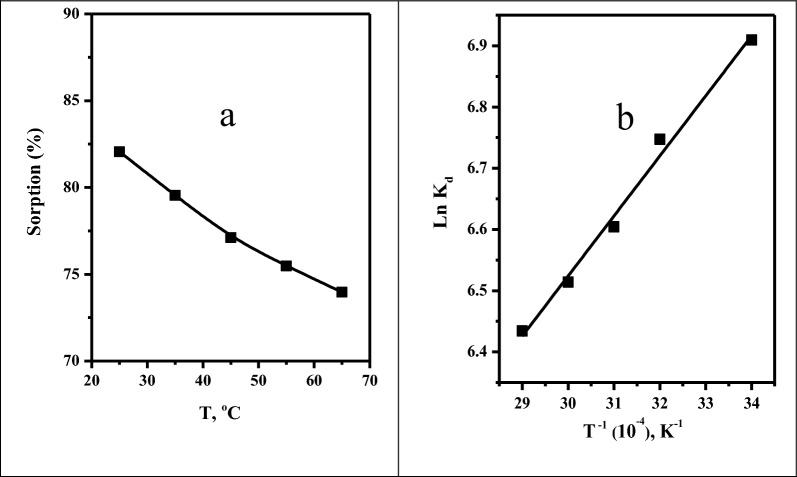
Effect of **a** temperature on the sorption efficiency **b** the thermodynamic parameters of Se(IV) ([Se(IV)] = 100 mg L^−1^, Dose = 0.10 g, pH = 3.0, t = 10.0 min, V = 5 ml, T = 25 ºC) from aqueous solutions

Figure 8a illustrates the impact of temperatures ranging from 25 to 65 °C on the sorption percentage of selenium from aqueous solutions. It is demonstrated that as the temperature increases, the adsorption percentage decreases gradually. Using the slope and intercept of the linear plot of Ln K_d_ against T^−1^ shown in Fig. 8b, the thermodynamic parameter (ΔG^o^) and the values of ΔH^o^ and ΔS^o^ were calculated. Table 5 includes the values of these parameters. From this table, it is noted that the sorption of selenium on Amberlite IRA-400 resin is spontaneous and available, as demonstrated by the negative values of ΔG_o_. The sorption process is exothermic nature, as indicated by the negative value of ∆H°. Moreover, the positive values of ΔS° suggested that the randomness of the system was increased.

**Table 5:** Parameters of thermodynamics for the sorption of selenium ions

Metal ions	T, K	ΔG^o^, k J mole^−1^	ΔH^o^, k J mole^−1^	ΔS^o^, J mole^−1^ K ^−1^
Se (IV)	298	− 17.024	− 8.15	29.78
	308	− 17.32		
	318	− 17.62		
	328	− 17.92		
	338	− 18.22		

#### Desorption and reusability studies

Selenium desorption from loaded Amberlite IRA-400 resin was also performed with several desorbing agents of different concentrations. The desorbing agents used included sodium acetate, nitric acid, and hydrochloric acid. According to Table 6 data and findings, 1.0 mol L^−1^ of nitric acid was used to produce a maximum Se(IV) desorbing of 96.40%. However, there is a slight difference in the results of using HCl and HNO_3_ as desorbing agents for the desorption of Se-ions from the resin. 0.5 mol L^−1^ HCl was used in the recycling process because the resin is present in the chloride form.

**Table 6:** Desorption of Se(IV) from loaded Amberlite IRA-400 resin using several desorbing agents of different concentration ranges*

Desorbing agent	Concentration (mol/L)	Desorption percent (%)
HNO_3_	0.10	88.90
	0.5	89.12
	1.0	96.40
HCl	0.10	91.42
	0.5	92.92
	1.0	93.66
Sodium acetate	0.5	89.45
	1.0	93.26

*V/m = 0.05 L/g, shaken time = 30.0 min, T = 25 ± 1 °C

Furthermore, the recycling process has numerous benefits for our environment. Recycling the materials we use decreases environmental pollution, helps preserve our health and the environment around us, and reduces the amount of waste that is buried in landfills.

**Fig. 9:**
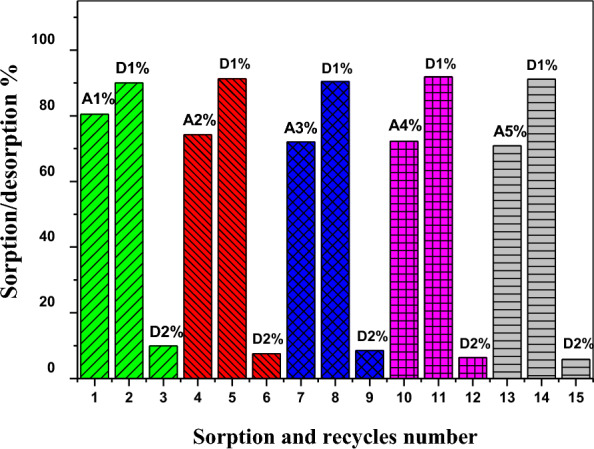
Reusability and stability of the resin for sorption of Se(IV) utilizing 0.5 mol L^−1^ HCl ([Se(IV)] = 100 mg L^−1^, Dose = 0.10 g, pH = 3.0, desorbing time = 30.0 min, T = 25 ºC)

In this study, five recycling processes were implemented for Se ions from the resin under the optimum conditions used in this study for adsorption–desorption on the resin as indicated in Fig. 9. From this figure, the loaded resin was nearly completely desorbed by 0.5 mol L^−1^ HCl after two cycles for each sorption stage. Where the first sorption reached 80.5% with two stages of desorption attained 99.9%. Then the second, third, fourth, and fifth sorption were 74.23, 72.88, 72.23, and 70.88% with their 2 cycles of desorption of 98.78, 98.90, 98.23, and 97.02%, respectively. These results also indicated the stability of the Amberlite IRA-400 resin after being used several times in the recycling process of Se(IV), where there is about a 10% difference between the first and fifth sorption processes.

#### Effect of competing ions

To determine the selectivity of Se(IV) from other competing ions using the amberlite IRA-400 resin, the sorption efficiency and the separation factor were studied and the results are tabulated in Table 7. From Table 7, it illustrated that the sorption % of Se(IV) is higher than of other competing ions and the best separator factor was between selenium and cobalt ions. This may be due to, (a) the ionic radius of Se^4+^ (0.5 A°) < Co^2+^ (0.745 A°) and (b) the electronegativity of Se^4+^ (2.55) > Co^2+^ (1.88) and for other competing ions as Se^4+^ (2.55) > Mo^6+^ (2.16) > V^5+^ (1.63) > Eu^3+^ (1.2) > Sr^2+^ (0.95) > Cs^+^ (0.79) as indicated in Table 7.

**Table 7:** Effect of interfering ions on K_d_ and separation factor (SF) by Amberlite IRA-400 resin at pH = 3.0 and V/m = 0.005 L/g

Competing ions	Ionic radius (A°)	Electronegativity	Sorption %	K_d__._(L/g)	Separation factor
					SF _Se__**/**__M_
**Se(IV)**	**0.5**	**2.55**	**94.56**	**0.087**	–
V(V)	0.59	1.63	55.87	0.006	14.5
Mo(VI)	0.65	2.16	65.23	0.009	9.67
**Co(II)**	**0.745**	**1.88**	**5.58**	**0.0003**	**290**
Eu(III)	0.947	1.2	23.58	0.0015	58
Sr(II)	1.12	0.95	19.75	0.0012	72.5
Cs(I)	1.67	0.79	11.94	0.0007	81.43

The highest sorption % and SF-values are bolded

#### Comparison with other adsorbents

In the comparison of Amberlite IRA-400 with other materials reported in Table 8, this adsorbent shows potential effectiveness for the removal of Se(IV) ions from aqueous solutions [20, 22, 23, 38–42].

**Table 8:** Comparison of Se(IV) adsorption by Amberlite IRA-400 with other materials

Adsorbents	pH	Q (mg/g)	References
Aluminum oxide coated sand	4.8	1.08	[20]
Iron coated granular activated carbon (Fe-GAC)	5	2.58	[22]
Nanocrystalline hydroxyapatite (FHAp)	5.0	5.51	[23]
Commercial hydroxyapatite (CHAp)	5.0	1.42	[23]
CS (chitosan)	5.0	1.35	[23]
FS (fish scale)	5.0	2.12	[23]
Magnetic Fe/Mn oxide nanomaterial	4.0	6.57	[38]
Magnetic nano particle-graphene oxide composites	6.0	23.81	[39]
Fe_3_O_4_ nanomaterials ( non microwave-assisted)	4.0	1.92	[40]
Fe_3_O_4 _nanomaterials (microwave-assisted)	4.0	2.38	[40]
Goethite	4	0.52	[41]
Hematite	4	0.39	[41]
modified rice husk	1.5	26.46	[42]
Amberlite IR-400	3	18.52	Current work

## Conclusions

In this research, the sorption of Se(IV) was performed using (Amberlite IRA-400) resin as an adsorbent under various batch adsorption experiments. To identify the Amberlite IRA-400 resin, several characterization techniques were employed, including FTIR, SEM, and EDX. The Langmuir isotherm and the pseudo-second-order kinetic model match the experimental results remarkably well. It was shown that Se(IV) has an experimental sorption capacity of 18.52 mg/g at pH 3.0. Moreover, the adsorption process was an exothermic, spontaneous reaction based on the thermodynamic properties (negativity of both ΔG° and ΔH°). In conclusion, the Amberlite IRA-400 resin exhibits great potential for selenium adsorption from aqueous solutions.

## Data Availability

All data generated or analyzed during this study are included in this published article.
